# The precision of ROTEM EXTEM is decreased in hypocoagulable blood: a prospective observational study

**DOI:** 10.1186/s12959-023-00468-5

**Published:** 2023-03-02

**Authors:** Lotta Sunnersjö, Henrik Lindström, Ulf Schött, Noa Törnquist, Thomas Kander

**Affiliations:** 1 Department of Intensive and Perioperative Care, Skåne University Hospital, 214 28 Malmö, Sweden; 2 The Medical Faculty, Lund University Sweden. Sölveg, 223 62 Lund, Sweden; 3 Department of Intensive and Perioperative Care, Skåne University Hospital, 221 85 Lund, Sweden

**Keywords:** ROTEM, EXTEM, Coefficient of variation, Hypocoagulability

## Abstract

**Background:**

The use of viscoelastic tests is becoming increasingly popular. There is a paucity of validation of the reproducibility of varying coagulation states. Therefore, we aimed to study the coefficient of variation (CV) for the ROTEM EXTEM parameters clotting time (CT), clot formation time (CFT), alpha-angle and maximum clot firmness (MCF) in blood with varying degrees of coagulation strength. The hypothesis was that CV increases in states of hypocoagulability.

**Methods:**

Critically ill patients and patients subjected to neurosurgery at a university hospital during three separate periods were included. Each blood sample was tested in eight parallel channels, yielding the CVs for the tested variables. In 25 patients, the blood samples were analysed both at baseline and after dilution with albumin 5%, as well as after being spiked with fibrinogen, simulating weak and strong coagulation.

**Results:**

In total, 225 unique blood samples were collected from 91 patients. All samples were analysed in eight parallel ROTEM channels, resulting in 1,800 measurements. In hypocoagulable samples, defined as those with values outside the normal reference range, the CV of CT was higher (median (interquartile range)) (6.3% (5.1–9.5)) than for normocoagulable samples (5.1% (3.6–7.5)), *p* < 0.001. CFT showed no difference (*p* = 0.14), while the CV of alpha-angle was higher in hypocoagulable samples (3.6% (2.5–4.6)) than in normocoagulable samples (1.1% (0.8–1.6), *p* < 0.001. The CV of MCF was higher in hypocoagulable samples (1.8% (1.3–2.6)) than in normocoagulable samples (1.2% (0.9–1.7)), *p* < 0.001. The CV ranges for the different variables were as follows: CT: 1.2%–37%, CFT: 1.7%–30%, alpha-angle: 0.0%–17% and MCF: 0.0%–8.1%.

**Conclusions:**

CVs for the EXTEM ROTEM parameters CT, alpha-angle, and MCF increased in hypocoagulable blood compared to blood with normal coagulation, confirming the hypothesis for CT, alpha-angle, and MCF but not for CFT. Furthermore, the CVs for CT and CFT were much higher than those for alpha-angle and MCF. The results demonstrate that EXTEM ROTEM results from patients with weak coagulation should be interpreted with the notion of limited precision and that procoagulative treatment, based only on ROTEM EXTEM, should be given with some caution.

**Supplementary Information:**

The online version contains supplementary material available at 10.1186/s12959-023-00468-5.

## Background

Viscoelastic haemostatic assays, such as rotational thromboelastometry (ROTEM) and thromboelastography (TEG), are valuable tools for guiding transfusion therapy and for the correction of different coagulopathies [1–3]. Given that it is common that ROTEM devices are placed outside the accredited clinical laboratory of hospitals close to the patients, it is very important to implement routines for quality assurance of the results [4]. According to the manufacturers recommendations internal quality control (IQC) should be performed continuously at least once a week and after every new installation to the device, maintenance or transportation. There are also international external quality controls (EQC) such as the ECAT Foundation where laboratories can voluntarily participate. The EQCs are based on lyophilized plasma dispatched to several laboratories at the same time, usually between 2–4 times a year. The results are then presented individually but can also be compared between the participating laboratories[4]. It should be noted that neither the internal nor the external quality controls use whole blood for analysing samples which is the normal approach for patient samples.

In 2011, the TEG-ROTEM working group concluded that thromboelastometry needs to be standardised and that more studies are required to validate this method and its reliability [5]. Since then, a handful of studies have evaluated the method using different sample types with various results. In one study containing both patients and healthy volunteers, the coefficient of variation (CV) for ROTEM clotting time (CT) in citrated whole blood was found to be 4%–12% [6]. The UK National External Quality Assessment Scheme (NEQAS) for blood coagulation used lyophilised plasma from 10 centres, demonstrating CV up to 15% in the EXTEM assay [7]. A retrospective investigation of ROTEM analyses showed no differences in CVs for CT, clot formation time (CFT), or maximal clot firmness (MCF) in patients with weak coagulation compared to patients with normal coagulation, but only duplicate measures were used [8]. To further investigate the precision of the EXTEM assay for citrated whole blood in the hypo-, normo-, and hypercoagulable states, we designed the present study in which the CV for eight parallel channels was examined. The aim was to measure CV for the ROTEM EXTEM assays CT, CFT, alpha-angle and MCF in blood with varying degrees of coagulation strength. We hypothesised that blood samples with weak coagulation would demonstrate increased CV for the ROTEM parameters compared to blood samples with normal coagulation.

## Methods

This was a prospective observational study performed at Skåne University Hospital, Lund, Sweden, with ethical approval from the Swedish Ethical Review Authority (Dnr 2010/482 and 2022–00,265-01). All participants gave signed informed consent before participation, and the manuscript was written in accordance with the STROBE-guidelines [9].

Patients were included during three separate periods: December 2018, June 2020 to June 2021, and February to April 2022. Both patients from the Intensive care unit (ICU) and patients subjected to neurosurgery were recruited during the first and second periods. The choice to include neurosurgery patients were only based on practical reasons. During the third period, only patients subjected to neurosurgery were recruited, and only in the third period were the blood samples modified in vitro. Patients undergoing elective neurosurgery were identified using the surgical planning software Orbit® (SYSteam Health & Care, Huskvarna, Sweden). As 3–5 patients underwent surgery at the same time, patients primarily subjected to intracranial tumour resections were selected. When two or more eligible intracranial tumour resections occurred on the same day, the patient whose surgery started first was included. In the case of two eligible surgeries starting simultaneously, the patient in the operating theatre with the lowest theatre number was included. On days when no intracranial tumour surgeries were scheduled, the patient whose surgery started first at the Department of Neurosurgery was included. Critically ill patients in the ICU were included at random, depending on staff being available to collect blood samples. Patients under the age of 18 and those unable to understand the information given in Swedish were excluded.

### Collection and handling of blood samples

All blood samples were collected through an arterial line in 2.7-ml tubes containing 0.109-M citrate tubes (Becton, Dickinson and Company Vacutainer Systems, Franklin Lakes, NJ, USA). For neurosurgery patients, blood was sampled before surgery and within three hours after the closure of the dura mater. Blood samples from critically ill patients were collected at inclusion and only once.

During the third inclusion period, blood samples were both analysed at baseline and modified in vitro with the purpose of investigating the performance of the ROTEM® device with both strong and weak coagulation. For the in vitro modification of the experiment, 0.27 ml fibrinogen (Fibryga®, Octapharma, Lachen, Switzerland) 20 mg/ml was added to 2.7 ml citrated blood, and 1.5 ml Albumin (Alburex®, CSL Behring, Pennsylvania, USA) 5% was added to 1.5 ml citrated blood, as previously described [10, 11].

### Rotational thromboelastometry

Each blood sample was simultaneously analysed in eight different channels: four in ROTEM® Delta (Pentapharm GmbH, Munich, Germany) and four in roTEG® (Pentapharm GmbH, Munich, Germany) with the same reagents being used as instructed from the manufacturer. The same reference values apply to the two devices and the results from the eight parallel ROTEM channels were used to calculate CV for the CT, CFT, alpha-angle and MCF parameters in each blood sample.

For classification of blood samples into hypo-, normo- and hypercoagulable states the manufacturers’ reference range was used. These were: CT (38–79 s), CFT (34–159 s), (alpha angle (63–83 degrees) and MCF (50–72 mm).

Before analyses, all samples were kept at rest in a pre-heated heating block at 37 °C for 20–30 min and analysed within three hours. The EXTEM assay was performed in accordance with the manufacturer’s instructions. This procedure was used for unmodified blood, blood spiked with fibrinogen, and blood diluted with albumin.

### Data acquisition

Data from the measurements were retrieved from the databases of the ROTEM devices and exported to an Excel (Microsoft Office 365, Microsoft Corporation Redmond, WA, USA) database for further analyses. Baseline patient data were retrieved from medical records (Melior™, Cerner Corporation, North Kansas City, Missouri, USA) and Orbit®.

### Statistics

The sample size was calculated based on the assumption that the CV for EXTEM CT in a hypocoagulable state would be 2% (units of percent) higher than the CV for EXTEM CT in a normocoagulable state, with a standard deviation of 4.5 (calculated from our previous unpublished results), a power of 95%, and a two-tailed alpha of 0.05. Given that the study was performed on three different cohorts (critically ill patients, neurosurgery patients and in vitro-modified samples), we expected that 1/4 of the samples would be classified as hypocoagulable, defined as a CT value above the normal reference range set by the manufacturer. Based on the above, the sample size was calculated to include a total of 216 blood samples (162 with normal CT values and 54 with high CT values). To allow for analysis failures and a lower ratio of hypocoagulable samples, the aim was to include 225 unique blood samples.

Values from analyses where the TEMograms were clearly incorrect were removed before calculating CVs. The data were exported to GraphPad Prism (version 9.3.1 for Windows, GraphPad Software, San Diego, California, USA) for statistical analysis.

The distribution of all variables was tested before choosing the appropriate statistical method. All non-parametric values were presented as medians with the interquartile ranges in parentheses and parametric values with mean and standard deviations. Numbers are presented with (%). The mean values of the eight channels for each variable and the corresponding CV were plotted in a scatterplot, and Spearman’s correlation was performed. The ROTEM variables were divided into groups based on whether the mean value of the eight channels for the variable was within, above, or below the manufacturers’ reference range. The CV for each measurement was calculated and compared between the groups. If the mean values for a variable could only be sorted into two groups, the Mann–Whitney U test was used for comparisons, and if the values could be divided into all three groups, a comparison between the groups was performed using the Kruskal–Wallis test with a post hoc Dunn’s test. To test whether the in vitro modification of the blood resulted in a hyper- or hypocoagulable state compared to baseline, Friedman’s test with a post hoc Dunn’s test was used. To test whether the values obtained from the four roTEG® channels were similar to those obtained from the four ROTEM®delta channels, XY plots and Bland–Altman plots were performed.

To address a potential problem with multiple testing, a modified Bonferroni correction was applied, and a *p*-value < 0.01 was considered significant.

## Results

In total, 91 patients were included. Of these, 16 were neurosurgery patients included in the first period with blood samples before and after surgery, 50 were critically ill patients included in the second period with single blood samples, and 25 were neurosurgery patients included in the third period. In this final period, blood samples before and after surgery were analysed at baseline and after adding fibrinogen or albumin, yielding six unique sets of blood samples per patient. The baseline characteristics are presented in Table 1. Out of the 232 total blood samples, seven were excluded due to pre-analytic mistakes, leaving 225 unique blood samples for analysis. All 225 samples were analysed in eight parallel ROTEM channels, resulting in 1,800 measurement points for the ROTEM assay. In total, 10/900 and 81/900 measurement points were excluded from the ROTEM®delta and roTEG® devices, respectively, due to clearly incorrect TEMograms.

**Table 1:** Baseline characteristics^a^

	*n* = 91 (100)
Age (years)	80 (50–69)
aPT-T^b^	26 (24–28)
Platelets (× 10^9^/L)	221 (169–271)
PT (INR)^c^	1.0 (0.9–1.2)
Male	55 (60)
Critically ill patients	50 (54)
Patients subjected to neurosurgery	41 (45)
ICU^d^ diagnosis	
− COVID-19	12 (13)
− Abdominal organ-related	10 (11)
− Perioperative	9 (9.9)
− Trauma	4 (4.4)
− Others	8 (8.8)
− Unknown	7 (7.7)
Neurosurgery operation diagnoses	
− Meningioma	11 (12)
− Frontal/temporal lobe tumour	11 (12)
− Pituitary tumour	6 (6.6)
− Others tumour	9 (9.9)
− Others – non-tumour	4 (4.4)
Neurosurgery operation details	
− ASA class I^e^	8 (8.8)
− ASA class II	25 (27)
− ASA class III	8 (8.8)
− Total bleeding (mL)	150 (60–200)
− Total IV fluids (mL)	1856 ± 458
− Operation time (min)	226 ± 70

^a^Numbers are presented with percentages, and continuous variables are presented with the median (interquartile range) or mean ± SD

^b^Activated partial thromboplastin time

^c^Prothrombin time (international normalized ratio)

^d^Intensive care unit

^e^American society of anesthesiologists (ASA) physical status classification system

**Fig. 1:**
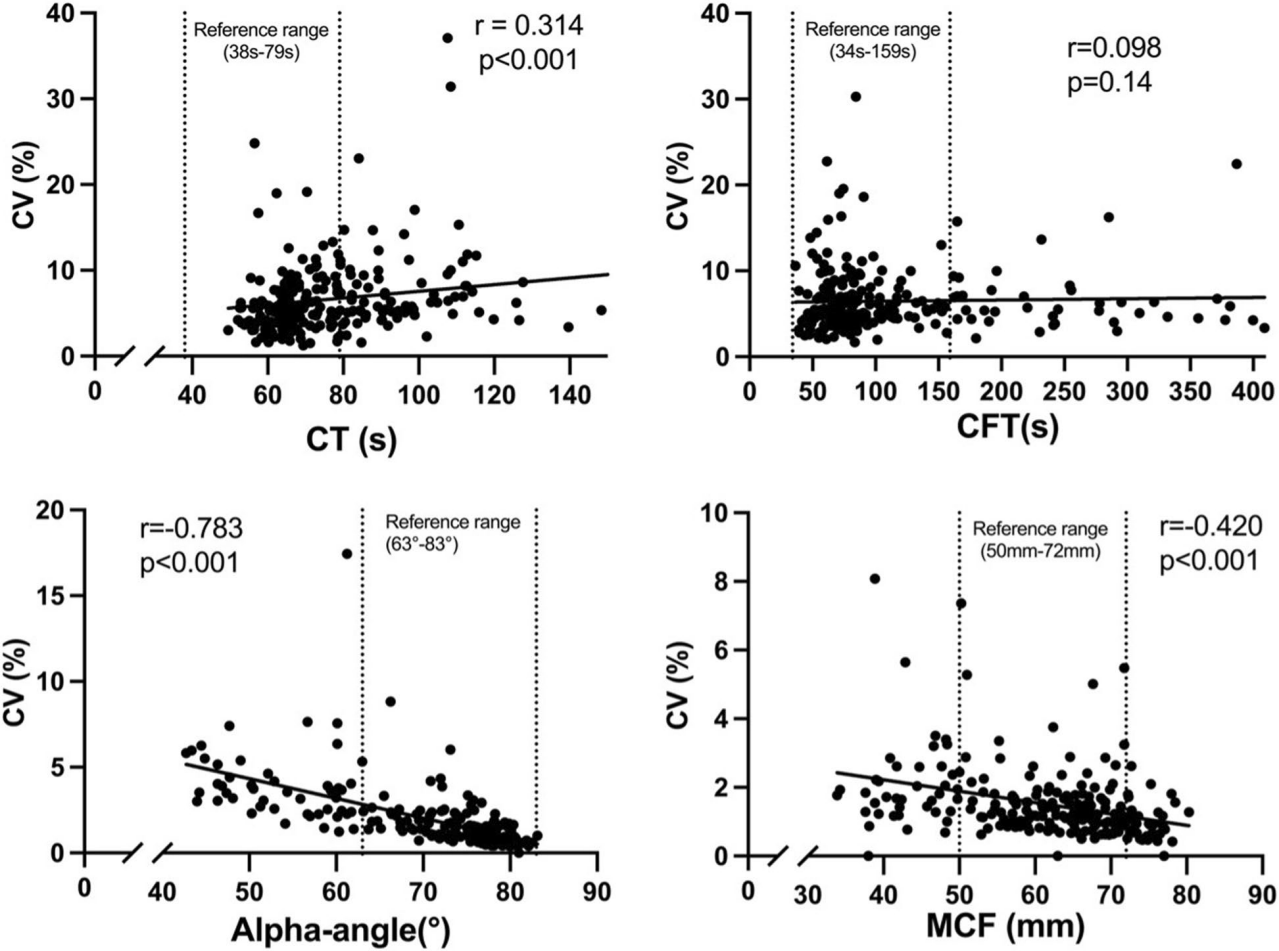
Correlations between mean values and corresponding coefficients of variation. Scatterplots Coefficients of variation (CV) plotted against mean values of the EXTEM assay from eight channels of clotting time (CT), clot formation time (CFT), alpha-angle, and maximum clot firmness (MCF), respectively. Two values in the CT scatterplot were outside the maximum range of the x-axis. Normal reference ranges are marked with vertical dotted lines. Spearman correlations with r-values are presented for each scatterplot

As two different ROTEM devices were used, scatterplots and Bland–Altman plots were performed to ensure coherence between the devices. The analyses demonstrated a strong correlation and consistency between the roTEG® and ROTEM®delta results, and the values from both devices were assessed as valid in aggregated analyses (Additional file 1). The desired effects of the in vitro modifications were confirmed, as fibrinogen spiking resulted in stronger coagulation and dilution with albumin resulted in weaker coagulation compared to the unmodified samples (Additional file 2). Significant correlations between mean value and the corresponding CV were demonstrated for CT (*r* = 0.314; *p* < 0.001), alpha-angle (*r* = 0.783; *p* < 0.001), and MCF (*r* = 0.420; *p* < 0.001), as shown in Fig. 1.

Based on the CT assay 80 (36) of 225 blood samples were classified as hypocoagulable. Out of these 61 (76) were unmodified samples. Based on the MCF assay 39 (17) were classified as hypercoagulable. Out of these 28 (72) samples were unmodified samples.

**Fig. 2:**
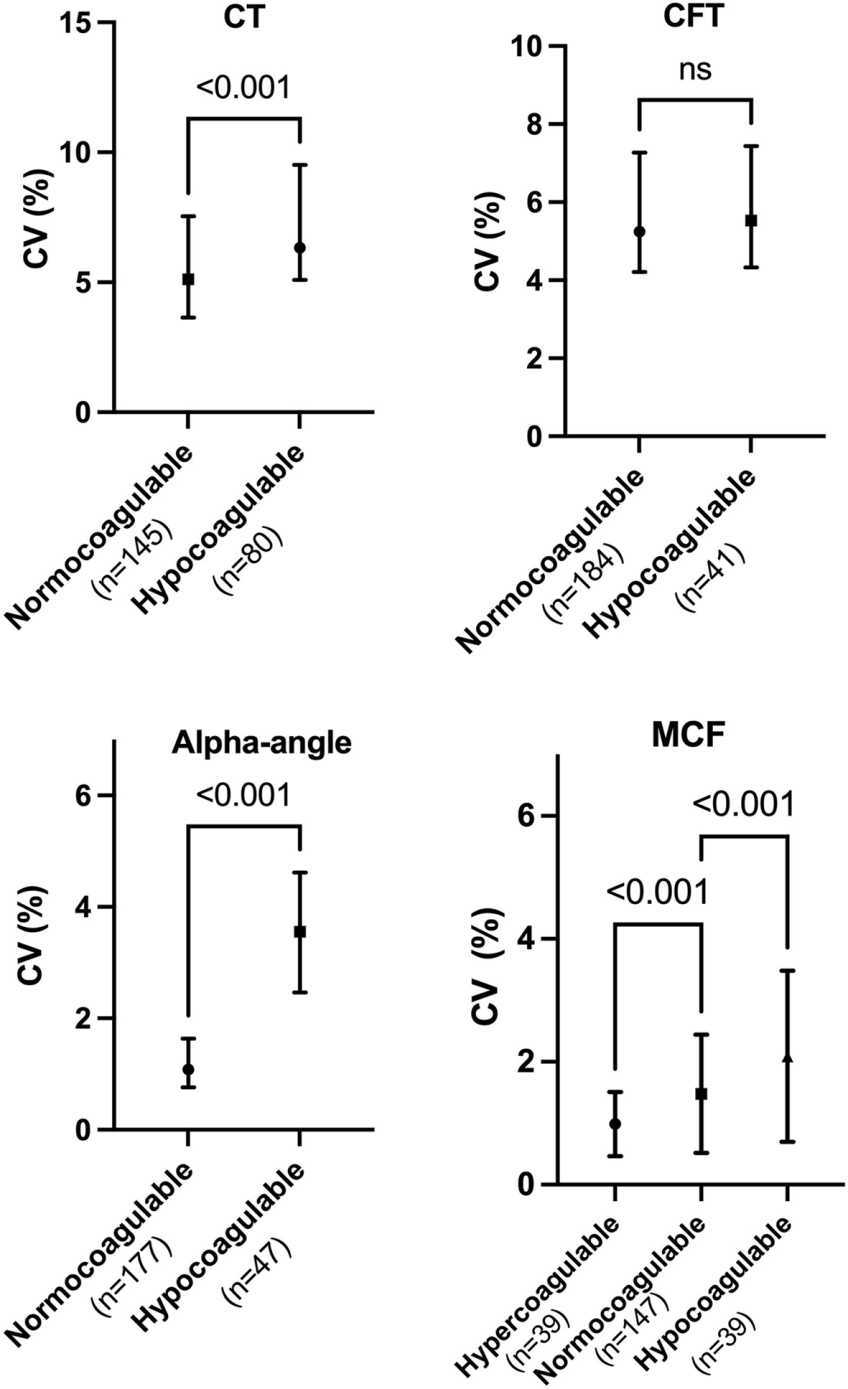
Comparison of the coefficients of variation in different coagulable states. Coefficients of variation (CV) from eight parallel channels for CT, CFT, alpha-angle, and MCF with medians and interquartile ranges. The groups were divided into normocoagulable, hypocoagulable, and hypercoagulable according to the manufacturer’s reference ranges for each of the ROTEM parameters. Mann–Whitney U tests for CT, CFT, and alpha-angle with p-values are outlined in the figure. Kruskal–Wallis and post hoc Dunn’s tests were used for MCF. One alpha-angle value was classified as hypercoagulable and excluded in the analysis. NS: not significant

To test the hypothesis that blood samples with weak coagulation would demonstrate increased CVs compared to samples with normal coagulation, comparisons between the CV of measurements with mean values within, below, and above the established reference ranges were performed. As demonstrated in Fig. 2, the CV of CT was higher in hypocoagulable samples (6.3% (5.1–9.5)) than normocoagulable samples (5.1% (3.6–7.5)), *p* < 0.001. For CFT, there was no significant difference (*p* = 0.14), while the alpha-angle CV was higher in hypocoagulable samples (3.6% (2.5–4.6)) than in normocoagulable samples (1.1% (0.8–1.6), *p* < 0.001. The CV of MCF was higher in hypocoagulable samples (1.8% (1.3–2.6)) than in normocoagulable samples (1.2% (0.9–1.7)), *p* < 0.001, and in hypercoagulable samples (0.9% (0.7–1.3)), the CV was lower compared to those in normocoagulable samples, *p* < 0.001.

**Fig. 3:**
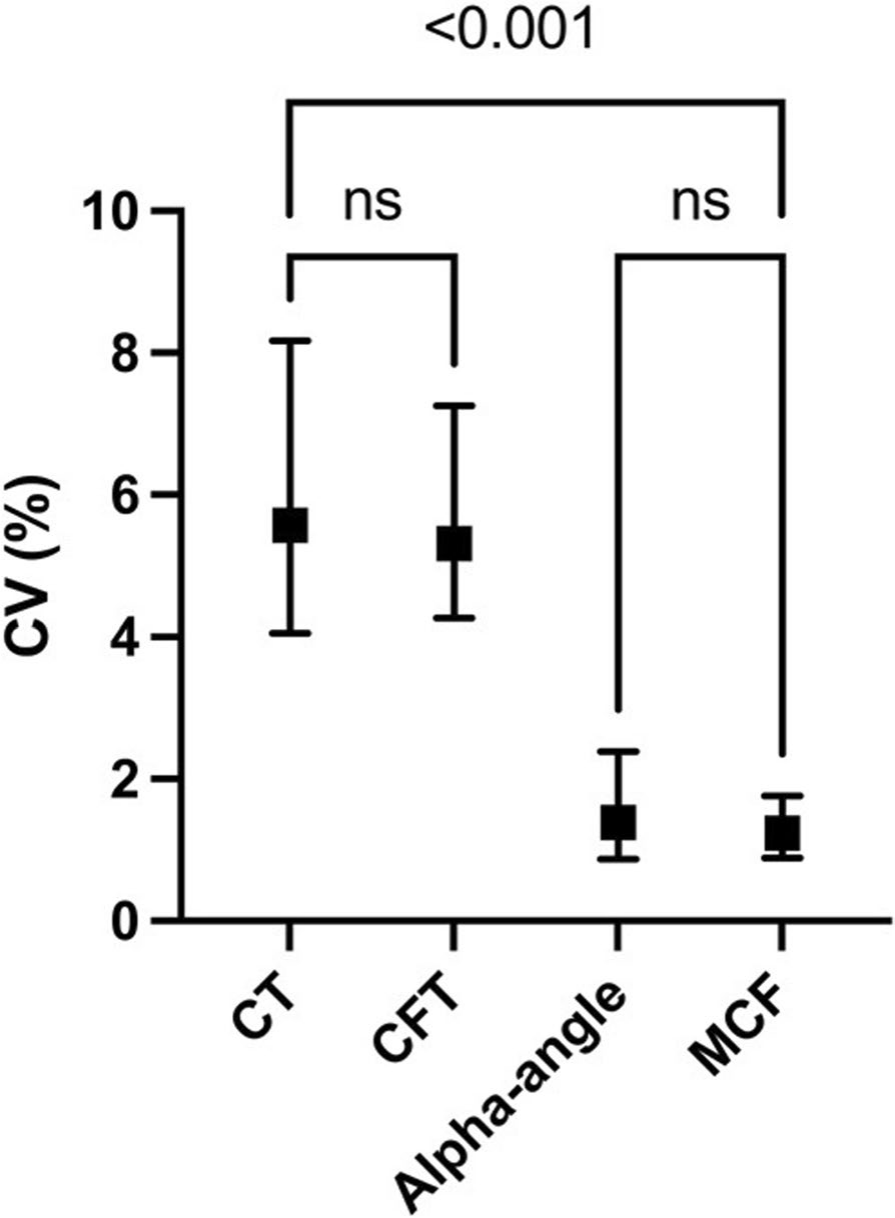
Comparison of the coefficients of variation between variables. The coefficients of variation (CV) for CT, CFT, alpha-angle, and MCF from the EXTEM assay. Median values with interquartile ranges are shown. Kruskal–Wallis and post hoc Dunn’s tests were used for all variables. Results not in the graph: CT vs alpha-angle: *p* < 0.001, CFT vs alpha-angle: *p* < 0.001, CFT vs MCF: *p* < 0.001

The CV for CT (5.6% (4.1–8.2)) and CFT (5.3% (4.3–7.3)) were higher than the CV for alpha-angle (1.4% (0.9–1.8)) and MCF (1.2% (0.9–1.8)), *p* < 0.001 (Fig. 3). The ranges of CVs for the different variables were as follows: CT: 1.2%–37%, CFT: 1.7%–30%, alpha-angle: 0.0%–17%, MCF: 0.0%–8.1%).

## Discussion

In this prospective observational study, we have demonstrated that CVs for the EXTEM ROTEM parameters CT, alpha-angle, and MCF increased in hypocoagulable blood compared to blood with normal coagulation, thus confirming the hypothesis for CT, alpha-angle, and MCF but not for CFT. Furthermore, the CVs for CT and CFT were considerably higher than those for alpha-angle and MCF. The results demonstrate that EXTEM ROTEM results from patients with weak coagulation should be interpreted carefully and with an awareness of the limited precision, especially for CT and CFT.

It should be noted that weak coagulation, as measured by the EXTEM ROTEM assay, is characterised by high CT and CFT values but low alpha-angle and MCF values. Hence, the slopes of the lines for CT, alpha-angle, and MCF in Fig. 1 (plotting CVs vs mean values) all demonstrate that values representing weak coagulation are associated with higher CVs, although the slopes lean in different directions. This is also confirmed in Fig. 2, where the CVs for CT, alpha-angle, and MCF are compared between hyper-, normo-, and hypocoagulable blood. Given that two study populations were included in the present study and that blood from the second cohort was also modified in vitro with the desired effects, the results are valid for a wide range of possible coagulable states compared to other studies where the analysis results were mainly within the reference range [6–8, 12–14].

Although the CVs for CT, alpha-angle, and MCF were higher in hypocoagulable blood, it should be noted that the overall CVs for alpha-angle and MCF were considerably lower than for CT and CFT (Fig. 3). This means that CT and CFT demonstrated the lowest precision and that a hypocoagulable state further impaired the precision of CT.

In a previous retrospective analysis of duplicate ROTEM measurements, overall good correlations between the samples were demonstrated in blood with normal coagulation [8]. However, the authors in that study also pointed out that some samples with results close to the limits of the upper or lower reference range did not correlate. These results are in agreement with the present study, where we performed eight measurements on each blood sample and confirmed that ROTEM EXTEM analyses of CT, alpha-angle, and MCF in hypocoagulable blood demonstrate significant imprecision. Furthermore, and concurrent with our study, Kitchen et al. showed that CT and CFT in the EXTEM assay were the most imprecise variables, while alpha-angle and MCF demonstrated more reproducible results [4]. This might indicate the need for careful interpretation of the results at a minimum and perhaps multiple measurements as well in certain circumstances.

As of today, there is no consensus of where CV for ROTEM EXTEM is regarded to be acceptable. However, it is an interesting finding that the CV differs between normocoagulable and hypocoagulable blood samples especially regarding the use of ROTEM algorithms. The ROTEM algorithms are valuable tools in the correction of acquired coagulopathies [15–17]. However, especially given the imprecision of CT in the hypocoagulable state, which was presented in the present study, we argue that the CT variable of the EXTEM assay should be used carefully in algorithms and that multiple samples should be considered before acting on the results. In several guidelines for the treatment of traumatic bleeding, ROTEM algorithms recommend the administration of prothrombin complex concentrates (PCC) when CT is prolonged. Given the low precision of CT, especially in the hypocoagulable state, and given that PCC is thrombogenic [18] and potentially dangerous, these recommendations can be questioned. As many ROTEM devices in clinical practice are placed outside the accredited clinical laboratory of hospitals it is very important to ensure regular quality checks along with calibration and instrument services. In this study, the devices were serviced and calibrated by the manufacturer just prior to the study.

We recognise the limitations of the present study. First, blood was collected at different time periods from different cohorts of patients and was modified in vitro in some cases. However improbably, this may have affected the results. Second, four different operators performed all analyses. Although all operators were carefully introduced and approved before patient recruitment, this may have affected the precision. Third, two different instruments were used (roTEG® and ROTEM®delta). Even though a good correlation between the results from the instruments was demonstrated, it cannot be ruled out that this affected the results. Fourth, the study investigated only the CVs for the ROTEM EXTEM assay, and the results may not apply to other available assays. Finally, some of the blood samples were modified in vitro. Even though > 70% of the blood samples classified as hypocoagulable in the CT assay were unmodified (innate hypocoagulable) as was > 70% of the hypercoagulable samples in the MCF assay, it cannot be ruled out that the modification of blood samples may have affected the results.

## Conclusion

In conclusion, the CVs for the EXTEM ROTEM parameters CT, alpha-angle, and MCF increased in hypocoagulable blood compared to blood with normal coagulation, confirming our hypothesis for CT, alpha-angle, and MCF but not for CFT. Further, CVs for CT and CFT were much higher than those for alpha-angle and MCF. The results demonstrate that EXTEM ROTEM results from patients with weak coagulation should be interpreted carefully, with an awareness of the limited precision.

## Supplementary Information

**Additional file 1:** Comparison between ROTEM® delta and roTEG®.

**Additional file 2:** In-vitro modified blood samples.

## Data Availability

The datasets used and/or analysed during the current study are available from the corresponding author upon reasonable request.

## Abbreviations

CV: Coefficient of variation
ROTEM: Rotational thromboelastometry
roTEG: Rotational thromboelastography
CT: Clotting time
CFT: Clot formation time
MCF: Maximum clot firmness
ICU: Intensive care unit
NEQAS: National External Quality Assessment Scheme
