# Supplementary material for: The precision of ROTEM EXTEM is decreased in hypocoagulable blood: a prospective observational study
Source: Thromb J. 2023 Mar 2;21:23. doi: 10.1186/s12959-023-00468-5 (PMC9978281; doi:10.1186/s12959-023-00468-5)
Supplement: Supplementary file 1 — Additional file 1: Comparison between ROTEM® delta and roTEG®. [file 12959_2023_468_MOESM1_ESM.docx]

**Additional file 1.** Comparison between ROTEM^®^ delta and roTEG^®^

**
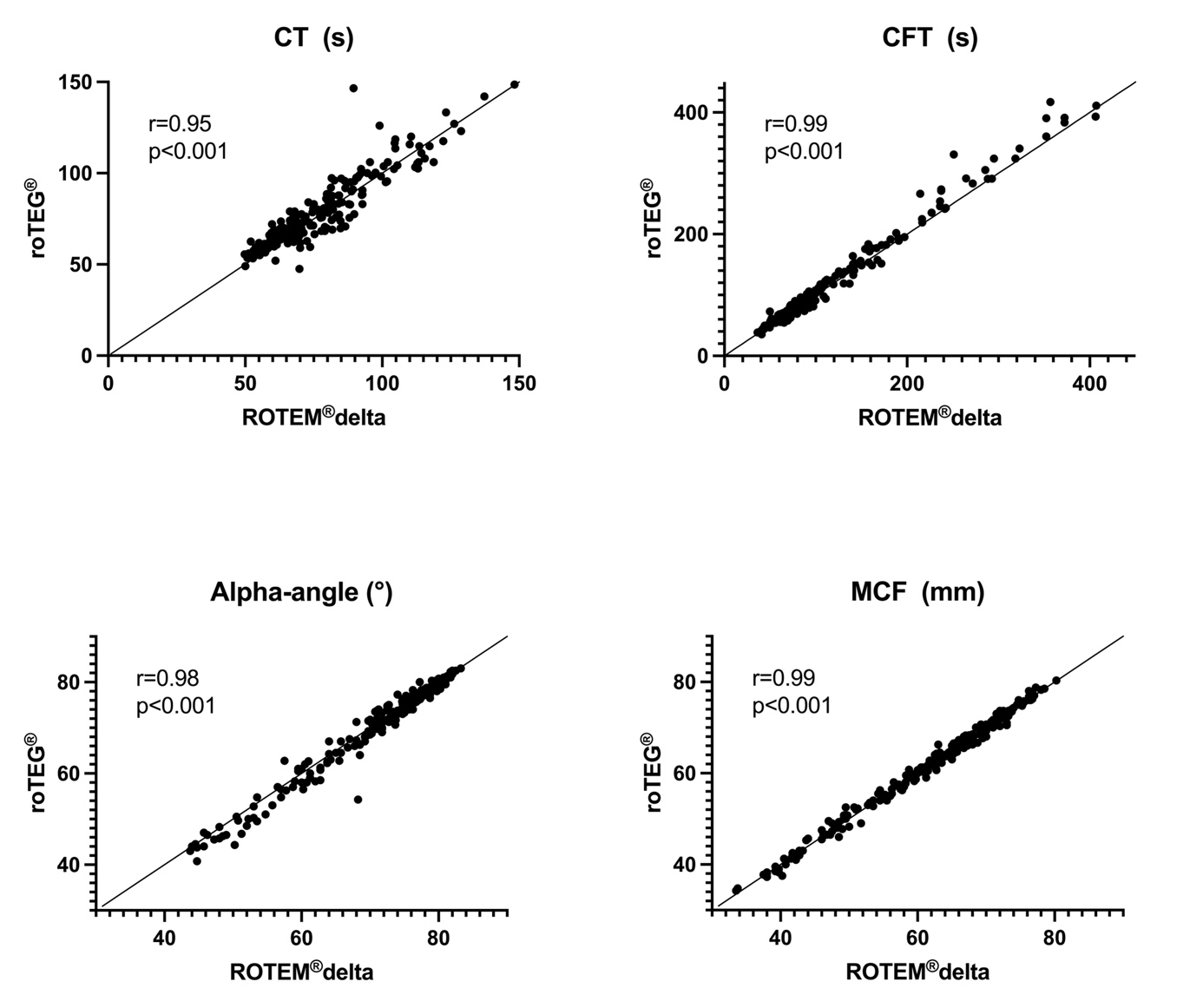
**

**Additional figure 1. roTEG^®^ and ROTEM^®^delta mean values are highly correlated.** Scatterplots where the mean values from four roTEG^®^ channels versus four ROTEM^®^delta channels are plotted. Clotting time (CT), clot formation time (CFT), Alpha angle, and maximum clot firmness (MCF). The filled lines represent x=y, i.e., a perfect match between the devices.


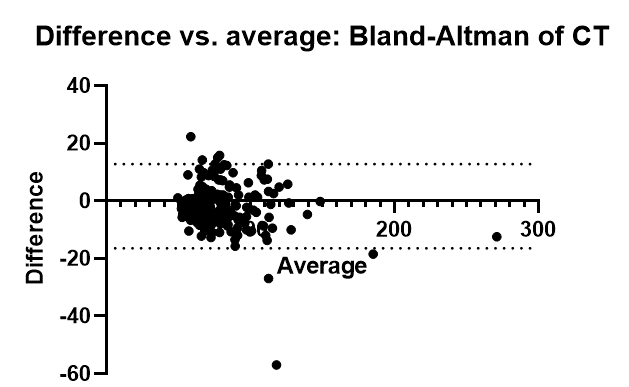

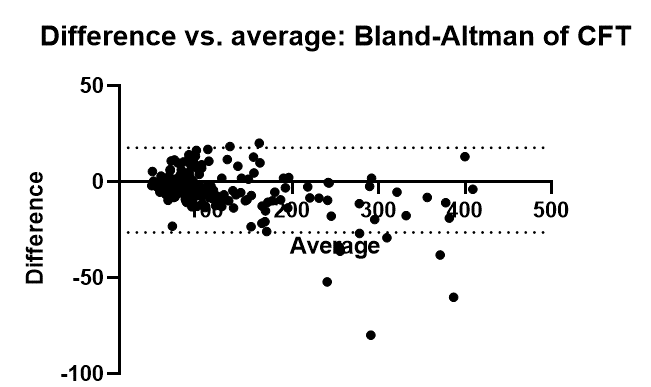

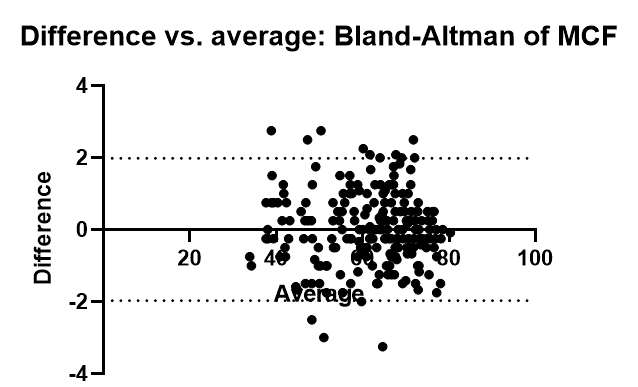

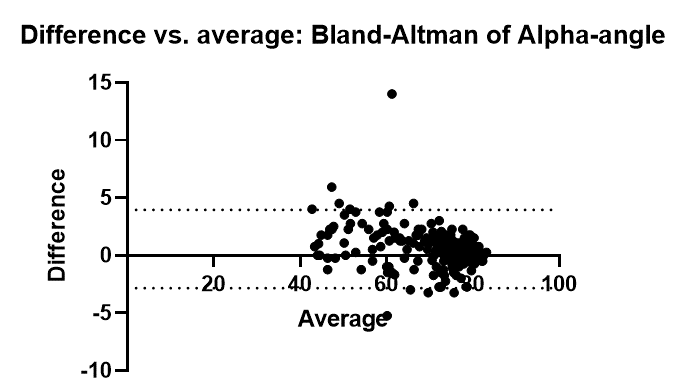


**Additional figure 2. Bland-Altman plots showing a high correlation between roTEG^®^ and ROTEM^®^delta.** Bland-Altman plots of the mean values of clotting time (CT), clot formation time (CFT), Alpha-angle, and maximum clot firmness (MCF) comparing results from four roTEG^®^ channels versus four ROTEM^®^delta channels to ascertain the correlation between the devices. The dotted lines indicate the 95% limits of agreement.
