# Supplementary material for: The precision of ROTEM EXTEM is decreased in hypocoagulable blood: a prospective observational study
Source: Thromb J. 2023 Mar 2;21:23. doi: 10.1186/s12959-023-00468-5 (PMC9978281; doi:10.1186/s12959-023-00468-5)
Supplement: Supplementary file 2 — Additional file 2: In-vitro modified blood samples. [file 12959_2023_468_MOESM2_ESM.docx]

Additional file 2. In-vitro modified blood samples^[[1]](#endnote-1)^.

| Parameter | Unmodified  Blood  (n=47) | Fibrinogen  modified blood  (n=47) | Albumin  modified blood  (n=47) | p-value^[[2]](#endnote-2)^ |
| --- | --- | --- | --- | --- |
| CT^[[3]](#endnote-3)^  (s) | 65  (60–68) | 61  (58–64) | 93  (82–111) | p<0.001 |
| CFT^[[4]](#endnote-4)^  (s) | 79  (57–98) | 61  (49–75) | 221  (153–290) | p<0.001 |
| Alpha-angle  (º) | 76  (70–78) | 79  (77–81) | 54  (48–61) | p<0.001 |
| MCF^[[5]](#endnote-5)^  (mm) | 63  (59–69) | 70.6  (67–75) | 46  (41–53) | p<0.001 |

1. For the matched comparison of the unmodified blood sample with in-vitro modified blood, two samples were omitted due to their matched pair that had previously been removed due to sample processing issues. Continuous variables are presented with median (interquartile range). [↑](#endnote-ref-1)
2. Friedmans test. P-value for all comparisons including Dunn´s multiple comparison test for Unmodified vs Fibrinogen and Albumin & Fibrinogen vs Albumin. [↑](#endnote-ref-2)
3. Clotting time [↑](#endnote-ref-3)
4. Clot formation time [↑](#endnote-ref-4)
5. Maximum clot firmness [↑](#endnote-ref-5)
